# Synergistic Effect of Thermoresponsive and Photocuring Methacrylated Chitosan-Based Hybrid Hydrogels for Medical Applications

**DOI:** 10.3390/pharmaceutics15041090

**Published:** 2023-03-29

**Authors:** Chun-Cheng Chen, Jie-Mao Wang, Yun-Ru Huang, Yi-Hsuan Yu, Tzong-Ming Wu, Shinn-Jyh Ding

**Affiliations:** 1 School of Dentistry, Chung Shan Medical University, Taichung City 402, Taiwan; 2 Department of Stomatology, Chung Shan Medical University Hospital, Taichung City 402, Taiwan; 3 Department of Materials Science and Engineering, National Chung Hsing University, Taichung City 402, Taiwan; 4 Institute of Oral Science, Chung Shan Medical University, Taichung City 402, Taiwan

**Keywords:** chitosan, gelatin, polyphenols, hydrogel, photocuring

## Abstract

The thermoresponsive drug-loaded hydrogels have attracted widespread interest in the field of medical applications due to their ease of delivery to structurally complex tissue defects. However, drug-resistant infections remain a challenge, which has prompted the development of new non-antibiotic hydrogels. To this end, we prepared chitosan-methacrylate (CTSMA)/gelatin (GEL) thermoresponsive hydrogels and added natural phenolic compounds, including tannic acid, gallic acid, and pyrogallol, to improve the efficacy of hydrogels. This hybrid hydrogel imparted initial crosslinking at physiological temperature, followed by photocuring to further provide a mechanically robust structure. Rheological analysis, tensile strength, antibacterial activity against *E. coli*, *S. aureus*, *P. gingivalis,* and *S. mutans*, and L929 cytotoxicity were evaluated. The experimental results showed that the hybrid hydrogel with CTSMA/GEL ratio of 5/1 and tannic acid additive had a promising gelation temperature of about 37 °C. The presence of phenolic compounds not only significantly (*p* < 0.05) enhanced cell viability, but also increased the tensile strength of CTSMA/GEL hybrid hydrogels. Moreover, the hydrogel containing tannic acid revealed potent antibacterial efficacy against four microorganisms. It was concluded that the hybrid hydrogel containing tannic acid could be a potential composite material for medical applications.

## 1. Introduction

Due to remarkable properties such as biocompatibility, biodegradability, tunable physicochemical properties, manufacturing versatility, and similarity to natural extracellular matrix (ECM), thermosensitive hydrogels with sol–gel transition at physiological temperature have attracted widespread interest in medical fields such as tissue engineering, drug delivery, tissue adhesives, and cell encapsulation [1,2,3,4,5,6]. More importantly, they can be molded in situ to the shape of the defect during minimally invasive surgery, which has the advantages of shortening the operation time and reducing postoperative pain. Taking dentistry as an example, periodontitis is a chronic inflammatory disease caused by periodontal pathogenic bacteria, which can lead to the formation of periodontal pockets, promote the growth of pathogenic microorganisms, and lead to tooth loss [7,8]. Bako et al. prepared poly-glutamic acid-based photopolymerizable nanocomposite hydrogels to investigate the release profiles of drugs for the treatment of the periodontal pockets [7]. Among hydrogel materials, highly biocompatible gelatin derived from collagen has been used as a carrier for drug delivery system and as a scaffold for tissue engineering in the type of membrane, fibrous particles, or hydrogel [9,10]. However, gelation of thermoreversible gelatin occurs at approximately 25 °C rather than physiological temperature [11,12]. Ideally, the injectable hydrogel material changes from a liquid state to a gel state when the temperature is raised to body temperature. As a result, lack of gelation at body temperature may limit the benefits of gelatin solution when injected in vivo, while lack of antibacterial ability is another clinical concern. In this regard, modifications are necessary to confer high bioavailability on gelatin.

Natural cationic polysaccharide chitosan, known for its antibacterial activity and mucoadhesive properties, has been widely used in medical application [5,13,14,15,16]. More specifically, the amino groups of chitosan allow modification with photocrosslinkable groups such as methacrylate group for photocuring [4,17,18] to improve its solubility and offer crosslinking. However, chitosan cannot enhance cell proliferation [19]. In contrast, gelatin can promote osteoblast activity and initiate specific integrin-mediated signal transduction between cells and materials, thereby promoting cell proliferation and differentiation [20]. Thus, the synergistic use of antibacterial chitosan and bioactive gelatin to construct solid-sate hydrogels at body-temperature was a feasible way to make use of their individual merits. Furthermore, chitosan/gelatin hybrid composites can be considered as simplified mimics of the natural ECMs composed of glycosaminoglycans and collagen [21,22]. Chitosan/gelatin hybrid composites have been indicated for healing of the nucleus pulposus in the intervertebral disc through minimally invasive implantation procedures [23] and have been developed as tissue adhesives for wound closure [24]. Osi et al. used 3D printing to prepare composites of methacrylate-modified chitosan and methacrylate-modified gelatin embedding nanohydroxyapatite for tissue engineering applications [4]. A photocrosslinked biopolymer of chitosan-methacrylate and gelatin was developed as a 3D-scaffold to mimic natural skin tissue [25].

The emergence of drug resistance urgently requires more effective non-antibiotic alternatives to treat bacterial infections. For this reason, naturally antimicrobial compounds have attracted great attention [26,27]. Natural polyphenols are organic compounds comprised of a phenolic ring bearing carboxyl and hydroxyl groups, which possess antioxidant properties and antimicrobial activities [27]. Phenolic compounds such as tannic acid and pyrogallol can be loaded or grafted onto the biomaterials to achieve clinical therapy. Sanandiya et al. fabricated a pyrogallol-functionalized chitosan-based hydrogel as a sealant for internal tissue treatment [28]. Singh et al. prepared a tannic acid-containing chitosan composite hydrogel for hemostatic efficacy [29].

To the best of our knowledge, there are limited data on chitosan-methacrylate and gelatin hybrid hydrogel gelled at the body temperature. To this end, we tuned the ratio of chitosan-methacrylate to gelatin to adjust the gelation temperature and time to endow initial thermal cross-linking at the physiological temperature, followed by photocuring to further provide a mechanical sturdy construct. In addition, another purpose was to study the effects of three phenolic compounds (tannic acid, gallic acid, and pyrogallol) on the physicochemical properties, mechanical properties, antibacterial activity, and cytotoxicity of methacrylated chitosan/gelatin composites with an aim at developing an effective medical hydrogel material.

## 2. Materials and Methods

### 2.1. Materials and Cells

**Figure 1:**
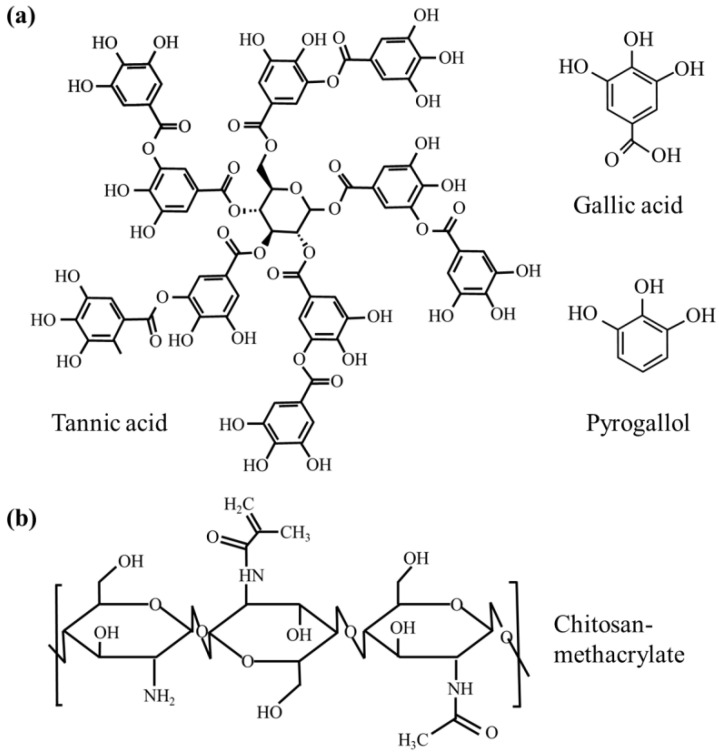
The chemical structure of (**a**) phenolic compounds (tannic acid, gallic acid and pyrogallol [30] and (**b**) CTSMA [31].

Chitosan (CTS) with 85% deacetylation (molecular weight < 5 kDa), type B gelatin (GEL), methacrylic anhydride (MA), fluorescamine (98%), dimethyl sulfoxide (DMSO), and lithium phenyl-2,4,6-trimethylbenzoylphosphinate (LAP; 95%) were purchased from Sigma-Aldrich (Aldrich, St. Louis, MO, USA). Gallic acid (GA; 3,4,5-trihydroxybenzoic acid monohydrate, 98%), pyrogallol (PG; 1,2,3-trihydroxybenzene, 99%), and tannic acid (TA, 95%) were obtained from Alfa Aesar (Heysham, UK), and their chemical structures are shown in Figure 1a. 

*Escherichia coli* (*E. coli*; ATCC 8739), *Staphylococcus aureus* (*S. aureus*; ATCC 25923), *Porphyromonas gingivalis* (*P. gingivalis*; A7436), *Streptococcus mutans* (*S. mutans*; ATCC 700610) strains, and L929 fibroblast cell line (RM60091) were obtained from the Bioresource Collection and Research Center (Hsinchu, Taiwan). Dulbecco’s modified Eagle medium (DMEM), fetal bovine serum (FBS), and penicillin/streptomycin solution were purchased from Gibco (Langley, OK, USA). The alamarBlue reagent was from Invitrogen (Grand Island, NY, USA). Bacto tryptic soy broth and Wilkins-Chalgren anaerobe broth were purchased from Becton Dickinson (Sparks, MD, USA) and Oxoid (Hampshire, UK), respectively. All reagents were of analytical grade and used as received without further purification.

### 2.2. Preparation of Chitosan Methacrylate

CTS was dissolved in 1% lactic acid and stirred for 1 h to form a 2 wt% solution. Afterwards, 4.74 mL of methacrylic anhydride (MA) was added to 80 mL of the solution and stirred vigorously for 1 h [32]. This mixture was then dispensed into a dialysis membrane and dialyzed against distilled water for 3 days, followed by freeze-drying at −20 °C to obtain a white spongy powder (CTSMA), which was stored at −20 °C until further use.

### 2.3. Preparation of Gel Films

CTSMA and GEL powders with a weight ratio of 3:1, 4:1, 5:1, and 6:1 (labelled as 3CMG, 4CMG, 5CMG, and 6CMG, respectively) were dissolved in 1% lactic acid and stirred for 1 h to obtain a uniformly mixed 6 wt% CTSMA/GEL precursor solution (CMG). 5 wt% phenolic acid powder (TA, GA, and PG) was added to the above CMG mixed solution, and then stirred for 1 h. The amount of 0.1% photoinitiator (LAP) was also added to the polyphenol-containing mixture and stirred for another 1 h. The hydrogel film was formed by light-curing the solution for 1 min using an LED light-curing device (Rolence, Taoyuan, Taiwan).

### 2.4. Determination of Degree of Substitution with Fluorescein

The degree of methacrylate substitution of free amine groups in CTS was detected using the fluorescamine assay [33]. First, 100 μL of 0.1 wt% CTS and CSMA in 1% lactic acid was mixed with 200 μL of fluorescein solution in DMSO according to the manufacturer’s guidelines. After the reaction, 200 μL of the solution was transferred to a 96-well microtiter plate and detected using a CLARIOstars microplate reader (BMG Labtech, Offenburg, Germany) at an excitation wavelength of 390 nm and an emission wavelength of 465 nm. The number of free amine groups was calculated using a glycine standard curve and compared with unsubstituted CTS to determine the final degree of substitution (DS).

### 2.5. Phase Composition and Thermal Behavior

Structural analysis of dried samples was performed using a high resolution X-ray diffractometer (XRD, Bruker D8 SSS, Karlsruhe, Germany) with Ni-filtered Cukα radiation operating at 40 kV, 100 mA, and a scan rate of 1°/min. The chemical structure was also examined by Fourier transform infrared spectroscopy (FTIR; Bruker Vertex 80v, Ettlingen, Germany) in transmittance mode with a spectral resolution of 1 cm^−1^ and a wavenumber range from 400 to 4000 cm^−1^. The powder was mixed with potassium bromide (Sigma-Aldrich) at a ratio of 1:100 and compressed into a pellet. The thermal behavior of CTS and CTSMA in air up to 250 °C at a heating rate of 10 °C/min was examined using a differential scanning calorimeter (DSC) (Mettler-Toledo 2-HT; Schwerzenbach, Switzerland). 

### 2.6. Surface Morphology

The surface morphology of the samples was analyzed using a field emission scanning electron microscope (FE-SEM; JEOL JSM-7800F, Tokyo, Japan) in lower secondary electron image (LEI) mode. The samples were subjected to critical point drying (E3100, Quorum Technologies, Laughton, UK) before JEOL Pt-plating (JEC-3000FC, Tokyo, Japan). In addition, optical images of the hydrogels were taken before and after photocuing using a Canon EOS M6 digital camera (Tokyo, Japan). 

### 2.7. Rheological Analysis

In order to determine the gelation temperature and gelation time, the rheological behavior of samples without photocuring was checked by a DHR-2 rheometer (TA Instruments, New Castle, DE, USA). G′ (storage modulus) and G″ (loss modulus) were obtained by dynamic temperature sweep and dynamic time sweep under 5% strain in the linear viscoelastic domain. In the dynamic temperature sweep, the frequency was set to 10 rad/s, and the temperature was raised from 25 °C to 45 °C at a rate of 2.5 °C/min. In the dynamic time sweep, the scan time was set to 300 s, and the sample was placed on the flat plate to preheat to 37 °C, and the time scan was performed after holding for 60 s.

### 2.8. Tensile Test 

Before and after drying at room temperature for 14 days, the samples were cut into rectangular pieces with 0.5 mm thick, 5 mm wide, and 10 mm long. Tensile strength was tested using a universal testing machine (EZ-SX, Shimadzu, Kyoto, Japan) at a loading rate of 10 mm/min. The formula for calculating the strength value is: tensile strength (MPa) = maximum fracture force (N)/cross-sectional area of the piece (mm^2^). Ten samples were measured to obtain the mean. 

### 2.9. Antibacterial Activity

The gel solution was dropped into a 48-well plate and irradiated with an LED light-curing device for 1 min to prepare samples, and then sterilized by 20 min of ultraviolet light. The strains used were Gram-negative *E. coli* and Gram-positive *S. aureus* grown in Bacto tryptic soy broth at 37 °C. In addition, periodontitis-associated bacteria such as Gram-negative *P. gingivalis* and Gram-positive *S. mutans* were also used to examine the antibacterial activity of the hybrid hydrogels, which were cultivated in Wilkins-Chalgren anaerobe broth under anaerobic conditions. Bacteria grown on the sample-free dishes were used as a negative control. About 500 μL of bacterial suspension per well containing 10^7^ CFU for 3, 6, and 12 h (short time) or 10^4^ CFU for 24 and 48 h (long time) was used. After the incubation time, samples were assessed for the antibacterial activity using alamarBlue. Plates were read at 570 nm with a reference wavelength of 600 nm in a BioTek Epoch microplate reader (Winooski, VT, USA). The bacteriostatic ratio (%) based on the absorbance of alamarBlue was calculated as follows: (absorbance of negative control–absorbance of samples)/absorbance of negative control × 100%. Three independent experiments were performed [27]. 

### 2.10. Cytotoxicity

Photocured sample preparation and sterilization for cytotoxicity were the same as those for antibacterial activity assessment. L929 fibroblasts (5000 cells/well) (BCRC RM60091, Hsinchu, Taiwan) were plated on the plates containing film samples and incubated for 12, 24, and 48 h. DMEM containing 10% FBS and 1% penicillin/streptomycin solution served as a negative control, and the medium containing 10% DMSO was used as a positive control. Cytotoxicity was determined using the alamarBlue assay [34]. Cell viability was normalized to the negative control based on absorbance measured by a BioTek Epoch spectrophotometer. Results were from three separate experiments. 

### 2.11. Statistical Analysis

One-way analysis of variance (ANOVA) and Duncan’s *post-hoc* tests were used to check for significant differences between means and variances, respectively. Statistical differences were set at a *p* value of <0.05.

## 3. Results

### 3.1. Chitosan Methacrylate

#### 3.1.1. Degree of Substitution

Fluorescamines can form highly fluorescent products after the reaction with primary amines within a few minutes, so it is used to detect amine-containing compounds, including peptides and proteins. This agent was therefore chosen to test the degree of substitution of amine groups in chitosan. The results of fluorescamine assay showed that 50% of the amine groups of methacrylic anhydride-modified chitosan were substituted. 

#### 3.1.2. Phase Composition

**Figure 2:**
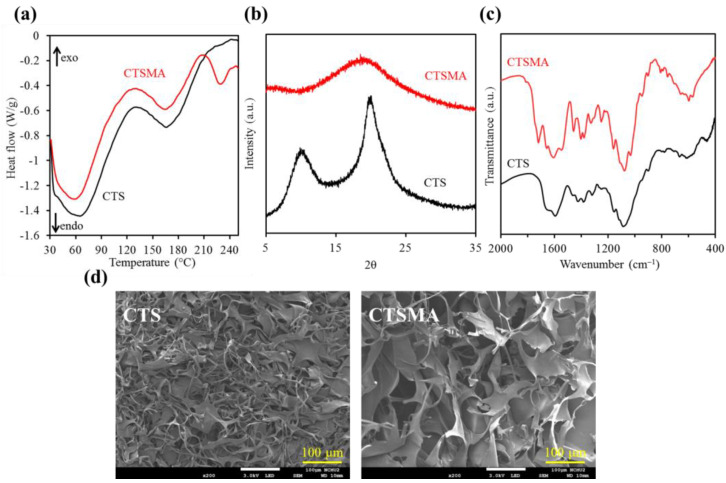
(**a**) DSC analyses, (**b**) XRD patterns, (**c**) FTIR spectra, and (**d**) SEM images of chitosan (CTS) and methacrylated chitosan (CTSMA) powders.

In the DSC results in Figure 2a, CTS has two endothermic bands at 64 °C and 165 °C. The former was due to the water evaporation in CTS, while the latter was attributed to the glass transition temperature (Tg) of CTS [35]. After modification with methacrylic anhydride, there was an additional and remarkable endothermic band around 230 °C, which was the glass transition temperature of MA in CTSMA, since the Tg of normal MA was about 250 °C [36]. The presence of CTS can shift the Tg to a lower temperature point, as described in previous studies [36,37].

The XRD patterns of unmodified CTS showed broad diffraction peaks at 2θ = 10.1° and 19.8° (Figure 2b), which were typical peaks of CTS with poor crystallinity [14,38]. In contrast, the diffraction intensity of CTSMA around 20° was broader and the peak around 10° disappeared, consistent with the previous findings [14]. This can be explained by breaking through the chemical modification of a large number of hydrogen bonds between the hydroxyl and amine groups within/intermolecular CTS, resulting in the formation of a low crystalline and/or amorphous phase [14,31].

The FTIR spectra of CTS before and after methacrylate modification are shown in Figure 2c. The bands at 1650 cm^−1^ (C=O), 1588 cm^−1^ (NH_2_), and 1069 cm^−1^ (C-O-C) were characteristic bands of unmodified CTS [25,31,39]. New CTSMA bands at 1538 cm^−1^ (N-H and C-N in amide II band) and 1379 cm^−1^ (amide III band) confirmed the methacrylation of CTS [25,31], while the band at 1718 cm^−1^ was ascribed to the grafting of methacrylate, as shown in Figure 1b.

#### 3.1.3. Surface Morphology

The SEM micrographs of CTS and CTSMA powders are shown in Figure 2d, which showed a spongy and porous structure-, similar to the previous study [31]. However, the flake area of CTSMA was larger than that of CTS, which was caused by the methacrylic anhydride modification as evidenced by the fluorescein assay. Differences in sample preparation might be another factor.

### 3.2. CMG Composites

#### 3.2.1. Surface Morphology

**Figure 3:**
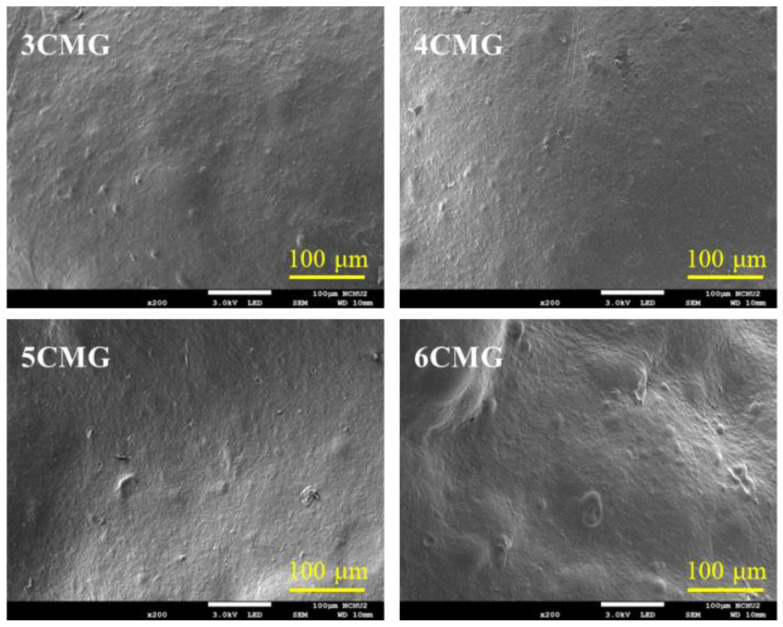
SEM micrographs of various CMG hybrid hydrogels.

The SEM images of various CTSMA/GEL (CMG) hybrid hydrogels are shown in Figure 3, revealing a smooth and non-porous appearance. It was shown that the blending of CTSMA and GEL was very homogeneous. However, it seems that agglomeration occurred on the surface of the higher content of CTSMA (6CMG).

#### 3.2.2. Gelation Temperature

**Figure 4:**
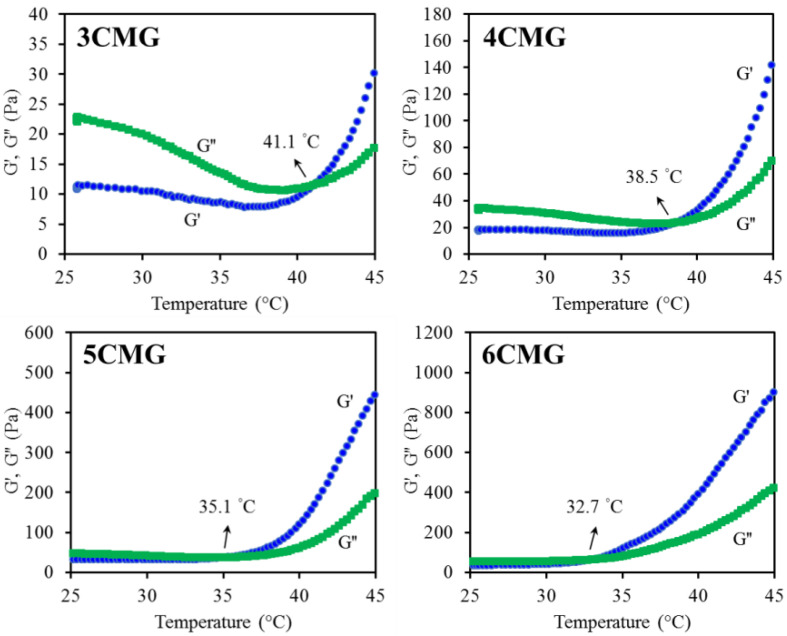
Dynamic temperature sweep analysis of various CMG hybrid hydrogels.

The viscoelasticity of the four formulations was evaluated to determine the gelation temperature by measuring the storage modulus (G′) representing the elastic behavior of the material and the loss modulus (G″) representing the viscous behavior. In general, a gel film precursor solution will behave like a solution, exhibiting a relatively smaller G′. However, the occurrence of gelation can promote the sudden increase in G′. Therefore, the gelation temperature refers to the temperature at which the gel precursor solution transforms from a solution to a gel, and is usually defined as the temperature at which G′ is equal to G″ [40], as shown in the representative graph of Figure 4. The increase in CSMA can lower the gelation temperature from 42 °C (3CMG) to 33 °C (6CMG). From the statistical result of Table 1, there was significant difference (*p* < 0.05) between four kinds of formulations.

#### 3.2.3. Gelation Time

**Figure 5:**
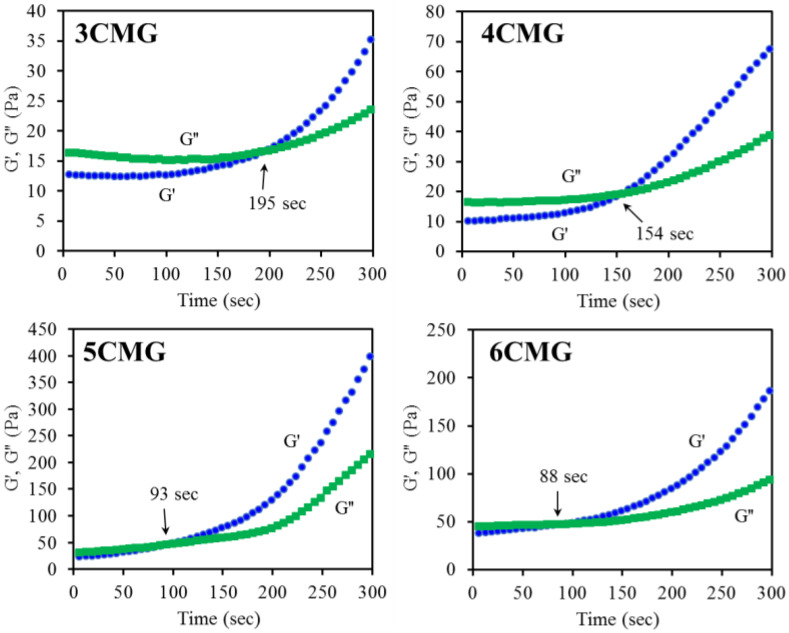
Dynamic time sweep analysis of various CMG hybrid hydrogels.

A dynamic time sweep of the gel film precursor solution was performed to detect the gelation time. Figure 5 is a representative graph showing a decrease in gelation time with increasing CTSMA, consistent with the observed trend in gelation temperature. It is evident that the gelation time values for 5CMG (91 s) and 6CMG (84 s) were significantly lower (*p* < 0.05) than for 3CMG (192 s) and 4CMG (159 s), as listed in Table 1. Based on gelation temperature and gelation time, of the four formulations 5CMG samples were further used for incorporation of three different phenolic compounds (TA, GA, and PG) for the subsequent cross-comparison and analysis.

#### 3.2.4. Photopolymerization

**Figure 6:**
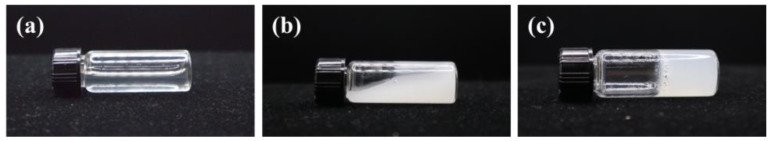
Optical images of (**a**) GEL, (**b**) 5CMG and (**c**) photocured 5CMG.

Figure 5 shows the optical images of GEL, 5CMG, and photopolymerized 5CMG at room temperature. Adding milky-white CTSMA to the transparent GEL liquid can significantly reduce the high fluidity of GEL (Figure 6a), showing a semi-solid hydrogel state (Figure 6b), and a completely non-flowing state after photocuring (Figure 6c).

### 3.3. Polyphenol Effect

#### 3.3.1. Composition and Morphology

**Figure 7:**
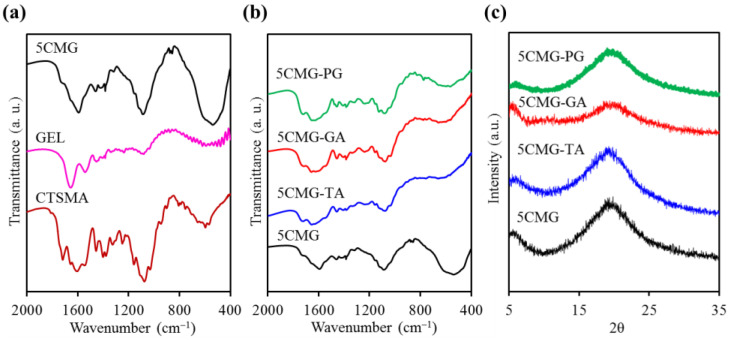
(**a**,**b**) FTIR spectra in transmittance mode of various samples and (**c**) XRD patterns of 5CMG with and without phenolic compounds (TA, GA, and PG).

**Figure 8:**
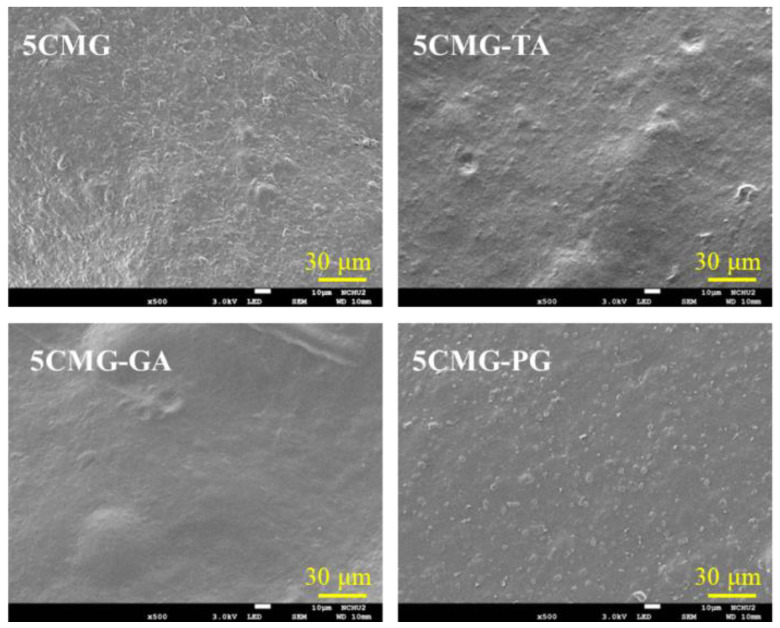
SEM micrographs of 5CMG samples with and without phenolic compounds.

Before unveiling the effect of phenolic compounds on the structure of the chitosan-based composites, the changes in 5CMG should first be elucidated. As shown in Figure 7a, the GEL structure has the bands at 1656, 1543, 1082, and 540 cm^−1^ attributed to CO stretching, NH bending, CH_3_ amide stretching, and COC group, respectively [41,42]. On the other hand, the 5CMG sample showed bands at 1721, 1655, 1593, 1088, and 542 cm^−1^, which were comprised of the characteristic bands of GEL and CTSMA with some overlapping bands. But the CTSMA band at 1606 cm^−1^ was shifted to a lower wavenumber at 1593 cm^−1^. When three different phenolic compounds were added to 5CMG, there appeared to be subtle differences in the bands despite the addition of small amounts of polyphenols (Figure 7b). The band around 1620 cm^−1^ was ascribed to the C=C stretching vibration of the aromatic ring [27], and the band at 550 cm^−1^ was broadened. In addition, 5CMG shifted from the band of 1088 cm^−1^ to a low wavenumber of 1075 cm^−1^, which may be due to the electrostatic and hydrophobic interactions between 5CMG and phenolic compounds. However, different phenolic compounds did not cause any band differences. Consistent with the FTIR trend, the XRD results indicated that a small amount of added phenolic compounds did not affect the phase evolution of 5CMG (Figure 7c). On the other hand, Figure 8 also shows that the addition of polyphenols did not lead to remarkable surface changes, all exhibiting smooth structures.

#### 3.3.2. Gelation Temperature and Gelation Time

When phenolic compounds were added, TA significantly increased (*p* < 0.05) the gelation temperature from 35.1 °C in the 5CMG group to 37.3 °C (Table 2). In contrast, addition of GA and PG had no statistically significant (*p* > 0.05) impact on the gelation temperature, which was in the range of 34.7–36.2 °C. Regarding the gelation time, the gelation time of the TA group (101 s) was comparable to that of the control group (91 s), and was significantly lower (*p* < 0.05) than that of the GA group (146 s) and the PG group (173 s).

#### 3.3.3. Tensile Strength

The tensile results showed that the addition of TA, GA, and PG increased the strength of the as-prepared membrane from the original 0.39 MPa to 0.56, 0.54, and 0.67 MPa, respectively (Table 2). It is reasonable that the strength of dry samples was significantly higher than that of wet samples because of the removal of liquid. After drying for 14 days, the presence of phenolic compounds enhanced the tensile strength of the hybrid films, which can be increased by up to 18.6 MPa due to the incorporation of TA. Statistical analysis also showed significant differences (*p* < 0.05) between 5CMG and 5CMG-TA and 5CMG-PG regardless of wet and dry conditions.

#### 3.3.4. Antibacterial Activity

**Figure 9:**
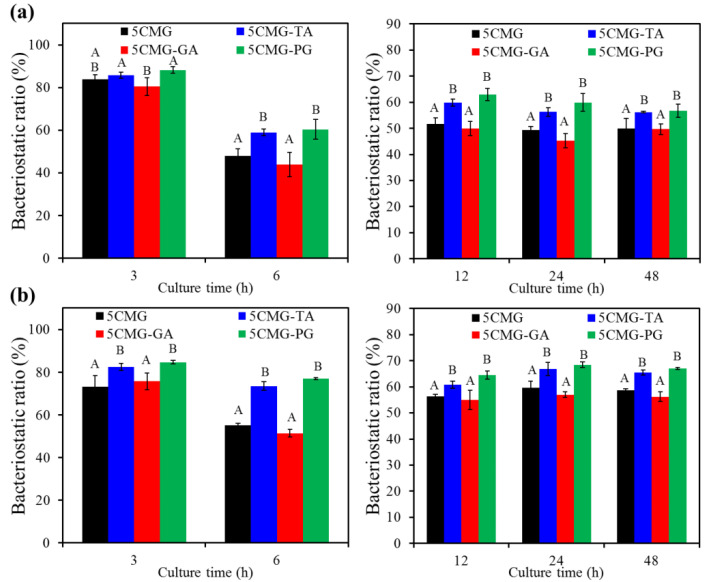
Bacteriostatic ratio of the various samples with and without polyphenols (TA, GA, and PG) against (**a**) *E. coli* and (**b**) *S. aureus* bacterial species after culture for short-term (3 h and 6 h) and long-term time points (12 h, 24 h and 48 h). Statistical comparisons were made between samples incubated at the same time point. Different capital letters indicated significant differences at *p* < 0.05.

Figure 9 shows the antibacterial efficacy of 5CMG with and without phenolic compounds against Gram-negative and Gram-positive bone-infection bacteria. The TA and PG groups were significantly (*p* < 0.05) higher than the GA group when inoculated with *E. coli* for short-term time intervals (e.g., 3 h and 6 h) (Figure 9a). After inoculation extended to 12–48 h, the TA and PG groups had significantly (*p* < 0.05) higher bacteriostatic ratios compared with the 5CMG and GA groups. Likewise, the TA and PG groups showed significantly (*p* < 0.05) higher antibacterial ability against *S. aureus* than the 5CMG control and GA groups at all seeding periods (Figure 9b). In addition, the addition of GA did not affect the antibacterial activity of 5CMG against Gram-negative and Gram-positive bacteria.

**Figure 10:**
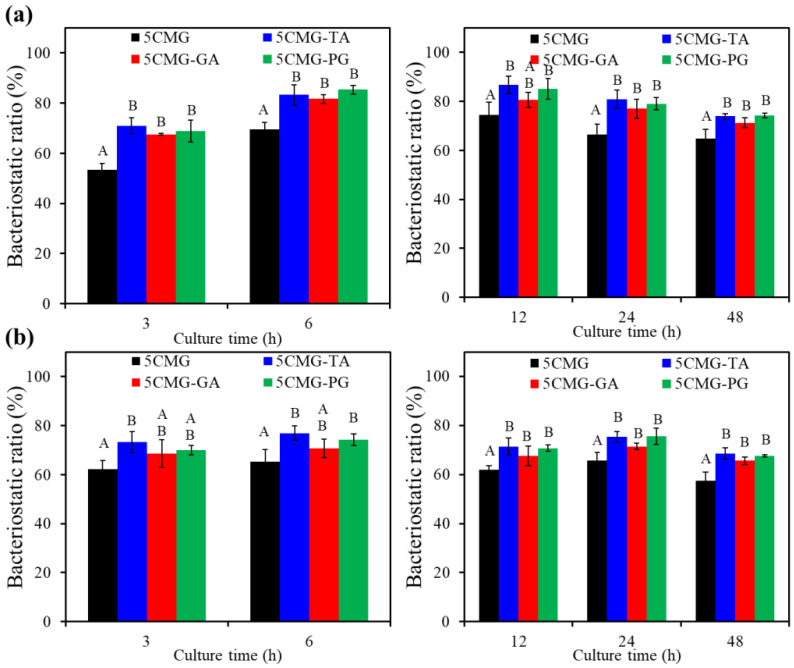
Bacteriostatic ratio of the various samples with and without polyphenols (TA, GA, and PG) against (**a**) *P. gingivalis* and (**b**) *S. mutans* bacterial species after culture for short-term and long-term time points. Statistical comparisons were made between samples incubated at the same time point. Different capital letters indicated significant differences at *p* < 0.05.

On the other hand, periodontal disease has become a serious public health problem worldwide due to its high prevalence and severe symptoms. The primary goal of periodontal treatment is the eradication of bacterial deposits. To expand the application of hydrogels developed in this study in the dental field, periodontitis-associated bacteria were used to evaluate the antibacterial activity of the materials. Figure 9 shows the results of inoculation of hybrid hydrogels with two periodontitis-associated bacteria. Regardless of the short-term or long-term culture, the bacteriostatic ratio of samples containing phenolic compounds against *P. gingivalis* (Figure 10a) and *S. mutans* (Figure 10b) remained at 70–80%, statistically significantly (*p* < 0.05) higher than the 5CMG control. Among the three phenolic compounds, the expression of bacteriostatic efficacy in the GA group was lower than that in the TA and PG groups, but there was no significant difference (*p* < 0.05).

#### 3.3.5. Cytotoxicity

**Figure 11:**
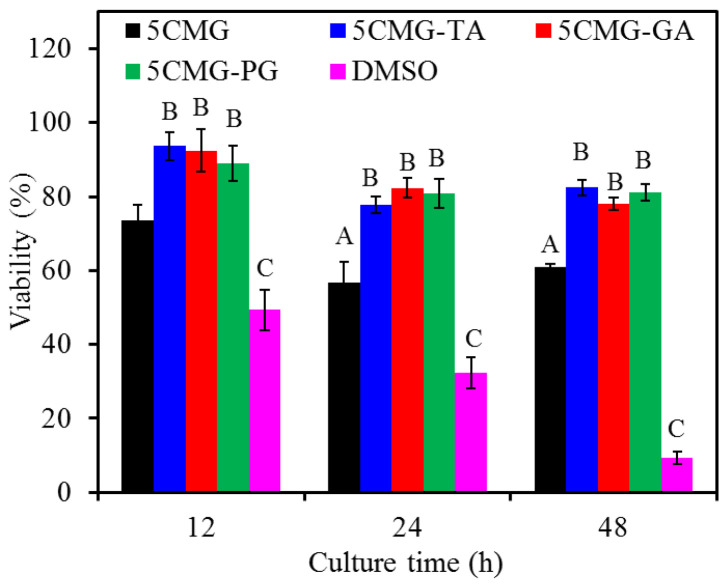
Cytotoxicity of various samples seeded with L929 cells at various time points. At the same cell culture time, different capital letters represented significant differences at *p* < 0.05.

With the increasing culture time, DMSO decreased the viability of L929 cells, indicating cytotoxicity (Figure 11). The 5CMG control revealed cell viability in the range of 60–70% during cell culture. In contrast, the presence of the three phenolic compounds significantly (*p* < 0.05) enhanced cell viability compared with the control at all culture time points. For example, L929 cells inoculated with samples containing the three phenolic compounds had approximately 80% viability after 48 h of culture. Moreover, the phenolic compounds-containing gel films did not show significant differences (*p* < 0.05) in cell viability at any culture time.

## 4. Discussion

The use of injectable or hand-moldable hydrogels should be a clinically practical approach [1,2], especially in structurally complex tissue defects such as osteoarthritis and periodontal pockets [7,10]. Although topical antibiotics are an ideal way to treat infections, effectively reducing drug doses and achieving adequate concentrations in areas of bacterial infection, the challenges posed by drug resistance remains unresolved [43,44]. In this study, different ratios of mucoadhesive methacrylated chitosan and bioactive gelatin were prepared, after which three phenolic compounds (TA, GA, and PG) were added to the hybrid hydrogel. DSC, XRD, FTIR, and SEM results showed clear evidence of methacrylate-grafted chitosan (CTSMA). Furthermore, FTIR spectra of CMG composed of CTSMA and gelatin by polymer blending revealed major vibrational bands associated with these two components, as well as some changes likely caused by multiple chemical interactions involving functional groups of the two polymers [25]. In addition, the morphology of the CMG hybrid hydrogel was very smooth and uniform, in agreement with a previous study [45].

In addition to imperative biocompatibility and tissue integration, an ideal hydrogel should also possess good handling to easily cover defect. Regeneration/repair materials should be in the physiological temperature ranges for optimal activity, i.e., close to oral temperature and body temperature. Thermosensitive hydrogels can turn their gelation behavior upon temperature change, especially when injected by using a syringe from an ambient temperature sol state to a physiological temperature gel state. Therefore, the gelation temperature and gelation time should be determined from a clinical point of view. In the rheological analysis for measuring gelation parameters, the crossover of G′ and G″ is regarded as the gelation point [40]. When the reaction temperature was lower than the gelation temperature, G′ was lower than G″, especially for hybrid hydrogels, such as 3CMG and 4CMG. Notably, the increased amount of CTSMA obviously improved the difference between G′ and G″ even at low temperatures. Moreover, as the reaction temperature increased, G′ increased sharply, and then G′ was higher than G″, indicating that the sample formed an elastic gel structure, and the molecular chains were not easy to move [46]. 

It is well-recognized that under normal conditions, the temperature of the mouth and body falls at 36–37 °C. The gelation temperature of the thermoresponsive gel used should be within this range, and the 5CMG group can roughly meet the requirement. Type B gelatin (isoelectric point = 5.0) used in this study is negatively charged [9] and can electrostatically complex with positively charged chitosan to from a polyionic blend [42,47], as evidenced by FTIR with lower vibration wavenumber [40]. Thus, as CTSMA increased, it can be speculated a decreased gelation time and gelation temperature. Morphology also confirmed agglomeration on surfaces with higher CTSMA content. The gelation time is defined as the time elapsed for the G′ of a gelled sample to be greater than its G″ at a given temperature [40]. Due to the principle of time-temperature superposition, the lower the gelation temperature, the shorter the gelation time [48]. Based on the above results, the thermoresponsive behavior and thermogelling mechanism of current hydrogels can be speculated as follows. CTSMA molecules with amino groups were protonated in acidic solution at room temperature, and electrostatic interactions occurred between the positively charged amino groups of CTSMA and the negatively charged amino acid residues of GEL [46,49]. On the other hand, the carboxyl groups of GEL can form hydrogen bonds with the amino groups in CTSMA, which may exist in the form of GEL-COO⋯H⋯NH_2_-CTSMA. At the same time, CTSMA/GEL (CMG) was rich in polar hydrophilic groups such as amino groups, carbonyl groups, and hydroxyl groups, and can also form hydrogen bonds with water molecules. When the temperature was raised to physiological temperature, the electrostatic attraction can reduce the electrostatic repulsion, causing attractive hydrophobic and hydrogen bonds [40]. In addition, Chang et al. found that increasing the temperature can increase the internal energy and break the hydrogen bonds between chitosan and water, which in turn made the hydrophobic chitosan chains tend to aggregate and gel [46]. In short, these factors caused the solution to turn into a hydrogel when the temperature was raised. In addition to physical thermal gelation, photocrosslinking of CTSMA was also used for synergistic gelation at physiological temperature [4], as evidenced by optical images. 

As for the phenolic compound effect, the gelation time of the three phenolic compounds varied in the following order: TA < GA < PG. This can be explained by the fact that TA (1701) had a higher molecular weight than GA (170) and PG (126), which led to molecule aggregation. More importantly, TA and PG brought the gelation temperature to about 36–37 °C, but the former had a shorter gelation time. 

By taking advantage of sequential thermo- and photo-cross-linking methods, the initial shapes of the film formed upon thermally induced gelation can be preserved, and the mechanical strength can be significantly enhanced by photo-cross-linking immediately after spreading onto body tissues. On the other hand, the mechanical properties of methacrylated chitosan/gelatin can be improved by phenolic crosslinking, supporting the stability of the formed structures. FTIR results indicated that electrostatic and hydrophobic interactions may occur between 5CMG and phenolic compounds. Indeed, the introduction of phenolic compounds such as TA in hybrid hydrogels may increase their application opportunities in biomedical applications by enhancing the unique properties. As suggested by Riveroa et al. [50], this is because of the reinforcement effect of the crosslinker throughout the biopolymer matrix. Given that phenolic compounds are a crosslinker due to the formation of hydrogen and electrostatic interactions [51], Sionkowska et al. found that the addition of TA to chitosan facilitated the crosslinking complex reaction [35]. GA and PG may play a similar reinforcing role. Phenolic compounds can covalently bind to amino group of chitosan [52]. It is reasonable that the dry process could promote the hardening of soft hydrogels due to the removal of water.

It is worth concerning whether there was compromise or synergy between CMG and phenolic compounds in terms of cytotoxicity or antibacterial activity. In the case of cytotoxicity, the addition of phenolic compounds improved the CMG response to L929 cell viability, increasing to levels of 80% at all culture time points. Cell viability greater than 70% is considered non-cytotoxic according to the specification of ISO 10993-5. According to the literature [53], phenolic compounds have been shown to stimulate the osteoblast growth.

The antibacterial property of hydrogel is an important clinical index. The antibacterial activity of the chitosan-based hydrogel is due to ionic interaction between its positive charges and negatively charged components on the bacterial cell wall, causing hydrolysis and leakage of intracellular electrolytes resulting in the microbial death [29]. From the results of statistically significant differences between the 5CMG-based hydrogels, it can be inferred that the presence of TA and PG enhanced the antibacterial ability against the tested bacterial strains. The hydroxyl groups in phenolic compounds are thought to cause antimicrobial action because these groups alter the cell membrane of bacteria, disrupting membrane structures and causing leakage of cellular components [26]. Dorman and Deans also reported that electronegative compounds reacted with vital nitrogen components such as proteins and nucleic acids to inhibit microbial growth through electron transfer [54]. Among the three phenolic compounds, the GA-containing hydrogel did not enhance the antibacterial activity against *E. coli* and *S. aureus*. This may be because the COOH functional group in GA played a key role in offsetting the NH2 functional of chitosan compared with PG and TA [27]. However, the presence of GA could enhance the antibacterial ability of the methacrylated chitosan-based hybrid hydrogels against *P. gingivalis* and *S. mutans*, which should be due to different bacterial strains. Overall, the synergistic efficacy of CMG and phenolic compounds can confer more potent antibacterial activity.

In this study, the hydrogel-based composites were first physically crosslinked by thermal gelation and then covalently photocured to form a stable structure. Methacrylated chitosan-based hydrogels can be formed into the desired shape of the surrounding tissues/organs for 3D conformal therapy, and can also be applied to inaccessible sites using a syringe. In addition, this photocurable hydrogel can also be used as a bioink for 3D printing, a barrier membrane for periodontal therapy, a scaffold for bone tissue engineering, a carrier for drug delivery, and a dressing for wound closure.

## 5. Conclusions

Thermoresponsive and photocurable methacrylated chitosan-gelatin hybrid hydrogels were modified by phenolic compounds to endow these hydrogels with unique functions. The composite with methacrylated chitosan/gelatin ratio of 5/1 had promising gelation temperature and gel time when TA was included. The addition of TA and PG to methacrylated chitosan-gelatin hydrogels significantly enhanced the antibacterial activity against four bacterial strains. More importantly, the added phenolic compounds can effectively improve the cell biocompatibility of the hybrid hydrogel. Considering the gelation behavior, tensile strength, and antibacterial ability, the hybrid hydrogel containing TA may be a potential composite material for medical applications. Further studies, such as the biodegradation and dose-dependent behavior of phenolic compounds are needed to ensure the application of potential hydrogels.

## Figures and Tables

**Table 1:** Gelation temperature and gelation time of CMG hybrid hydrogels.

Sample Code	CTSMA to GEL	Gelation Temperature (°C)	Gelation Time (s)
3CMG	3:1	41.3 ± 0.8 ^A^	192 ± 9 ^A^
4CMG	4:1	38.4 ± 1.0 ^B^	159 ± 8 ^B^
5CMG	5:1	35.1 ± 0.7 ^C^	91 ± 10 ^C^
6CMG	6:1	32.8 ± 0.3 ^D^	84 ± 20 ^C^

Different capital letters in superscript (A–D) showed statistically significant differences at *p* < 0.05.

**Table 2:** Polyphenol effect on gelation temperature, gelation time and tensile strength of 5CMG hydrogels.

Sample Code	Gelation Temperature (°C)	Gelation Time (s)	Tensile Strength (MPa) as-Prepared 14-d Drying	
5CMG	35.1 ± 0.7 ^A^	91 ± 10 ^A^	0.39 ± 0.01 ^A^	15.2 ± 1.4 ^A^
5CMG-TA	37.3 ± 0.8 ^B^	101 ± 9 ^A^	0.56 ± 0.07 ^B^	18.6 ± 1.7 ^B^
5CMG-GA	34.7 ± 1.2 ^A^	146 ± 3 ^B^	0.54 ± 0.12 ^A,B^	17.0 ± 1.6 ^C^
5CMG-PG	36.2 ± 0.7 ^A,B^	173 ± 18 ^C^	0.67 ± 0.08 ^B^	17.5 ± 1.7 ^B,C^

Different capital letters in superscript (A–C) showed statistically significant differences at *p* < 0.05.

## Data Availability

Not applicable.
